# Rapid Detection of Glucose on Nanostructured Gold Film Biosensor by Surface-Enhanced Raman Spectroscopy

**DOI:** 10.3390/bios11020054

**Published:** 2021-02-19

**Authors:** Cheng-Ju Sung, Szu-Han Chao, Shih-Chieh Hsu

**Affiliations:** Department of Chemical and Materials Engineering, Tamkang University, New Taipei City 25137, Taiwan; ks4080612345@yahoo.com.tw (C.-J.S.); ssuhanchao@gmail.com (S.-H.C.)

**Keywords:** SERS, AuFON, cyclic voltammetry, Rhodamine 6G, glucose, biosensor

## Abstract

In this report, we summarized our development of biosensors for Rhodamine 6G and in vitro glucose detection based on surface-enhanced Raman scattering technology. For the detection of both Rhodamine 6G and in vitro glucose, a nature-patterned substrate with gold films over nanostructures (NPS-AuFON) was used as the surface-enhanced Raman scattering sensor platform. The enhancement factor was calculated at 9 × 10^7^. In the processing of the substrate, cyclic voltammetry was used to form nano-gold particles under different conditions. The Rhodamine 6G and glucose detection were then achieved on this substrate. Furthermore, we combined the potentiostatic technique and electrochemical adsorption to best detect glucose in low concentrations. The glucose oxidation potential (100 mV) was used to capture glucose close to the surface of the NPS-AuFON. The quantitative detection of glucose in solution and in situ inspection were confirmed. Further, we determined that this surface modification technology can reach the goal of experiments set by the World Health Organization to judge whether or not a patient is a diabetic by detecting a glucose concentration of 11.1 mmol/L (mg/dL) at a minimum.

## 1. Introduction

In 1974, Fleischmann et al. [1] added pyridine molecules to 0.1 M of potassium chloride electrolyte to perform several redox reactions on a silver electrode. As a result, the Raman signal of a single layer of molecules was detected on the surface of that silver electrode. They speculated that this was caused by the redox treatment performed on the silver electrode in the experiment which caused the surface roughness of the silver electrode to increase, and subsequently it adsorbed more pyridine molecules. They found that when the number of pyridines increased, the overall Raman scattering intensity increased, resulting in the observation of the Raman signal in a single layer of molecules. This was the first reported phenomenon of Raman signals being amplified, and the so-called surface-enhanced Raman scattering (SERS). In 1977, Van Duyne et al. studied the adsorbed pyridine on a silver surface and extended its applicability to other nitrogen heterocycles and amines [2]. They also found that the intensity of the Raman spectrum increased by 10^5^ and claimed that redox could not fully explain the increase of the Raman signal, thus they mentioned the concept of an adsorption enhancement effect. With the further development of SERS, most people believed that SERS was caused by electromagnetic and chemical effects [3]. Due to the development of SERS technology, researchers were then able to qualitatively or quantitatively determine small molecules typically hard to observe. Therefore, in the mid-2000s, most researchers used Rhodamine 6G (R6G) as the standard analyte and silver or gold as its SERS substrate to test the detection limit and calculate its enhancement factor (EF) [4,5,6]. The SERS EF is the intensity ratio between SERS and normal Raman scattering for a given analyte normalized by the number of molecules probed. Furthermore, some researchers started to develop this technique into the field of biomedicine [7,8,9,10,11]. For example, Y. Zhao et al. (2008) used principal component analysis (PCA) combined with Raman spectroscopy, where the SERS spectrum clearly showed the signal difference between a variety of species [8]. It also demonstrated how to distinguish between non-viable cells and viable cells. Their results proved that SERS technology could classify different bacterial strains even when the Raman characteristic peaks of the analytes were similar.

Nowadays, an estimated 34.2 million people in the United States have diabetes (types I and II), accounting for 10.5% of the total population. An estimated 88 million Americans aged 18 years or older have prediabetes and a quarter of diabetic patients do not know that they have this disease [12]. In 1997, the total expenditure for diabetes diagnosis, monitoring, and treatment was US $44 billion [13]. From the statistics, we determined that blood glucose measurement has become crucial. Traditional electrochemical blood glucose measurement has mainly used the oxide obtained by a catalyst to learn patients’ blood glucose concentration. This method is fast and simple, so it is currently widely used. Nevertheless, the catalyst is unstable, so the blood glucose test paper with the catalyst on it has a very short lifetime, generally only three months. Deteriorated test paper makes mistakes because the catalyst is susceptible to environmental changes. To prevent these problems, we aimed to introduce the Raman scattering method as a replacement for the traditional electrochemical method.

As mentioned above, Raman scattering has been used in the field of biomedicine for some time. However, for the detection of low-concentration biomolecules, general Raman scattering has its detection limits. Therefore, SERS technology has been applied to enhance the Raman signals. SERS technology modifies the surface of the substrate (usually a thin film layer of precious metal) with nanoparticles, and then the object to be measured is placed on the substrate for Raman analysis. Gold or silver nanoparticles on the surface have a resonance effect, which can amplify the signal of the object so that it is more easily measured. As for SERS technology, the crucial technical factor is the EF value, which can generally reach the level of 10^6^–10^10^, as shown in the literature [4,5,6,14,15,16]. The key to SERS technology lies in the substrate that can increase the EF value. SERS substrates are usually made of nano-gold or -silver particles, with a diameter of 10–200 nm. Some process methods are used to make them arrange regularly [16]. When using Raman scattering to detect glucose, some researchers have used light analysis tubes, photonic crystal fiber, animals, and silver and gold as SERS substrates to detect glucose [17,18,19,20,21]. However, glucose cannot be easily adsorbed by precious metals. To ameliorate the situation, some studies used different self-assembled monolayers (SAM), such as 1-decanethiol (1-DT), (1-mercaptoundecyl-11-yl)tri(ethylene glycol) (Eg3), glucose oxidase (GOD), and horseradish peroxidase (HRP). Complex SAM, such as decanethiol (DT) and 6-mercapto-1-hexanol (MH), have also been adopted to capture glucose on the SERS substrate for detection [22,23,24,25,26,27,28].

Van Duyne et al. have done the most in-depth research of glucose detection using Raman spectroscopy [9,10,11]. However, the Van Duyne team mainly used SAM to grab glucose molecules for detection, rather than improving the surface of the SERS substrate. Although the SAM can effectively enrich glucose molecules on the surface of the substrate, its production process is quite complicated. In this way, the cost of the analytic equipment would increase. The steps are also cumbersome. It has other disadvantages, such as poor thermal and mechanical stability, decayed Raman signals, and reduced grasping efficiency (lifetime), along with the number of uses [29]. In order to solve these problems, our team avoided the use of SAM, developed a new type of substrate manufacturing and measurement technology, and found an optimal surface morphology through experiments to achieve economical and rapid detection of glucose in low concentrations. According to our experimental results, the detection limit reached the minimum level of 11.1 mmol/L (mg/dL) of glucose in a human body, which was issued by the World Health Organization (WHO) to distinguish healthy people from diabetic patients 2 h after eating [30].

## 2. Materials and Methods

### 2.1. Chemicals

All chemicals used were reagent grade or higher. All solutions were prepared with reverse osmosis (RO) water. Sulfuric acid (H_2_SO_4_) was purchased from Honeywell Fluka™. Sodium hydroxide (NaOH) was purchased from SHIMAKYU Chemical. Glucose (C_6_H_12_O_6_) and R6G were purchased from Sigma-Aldrich.

### 2.2. Substrate

**Figure 1:**
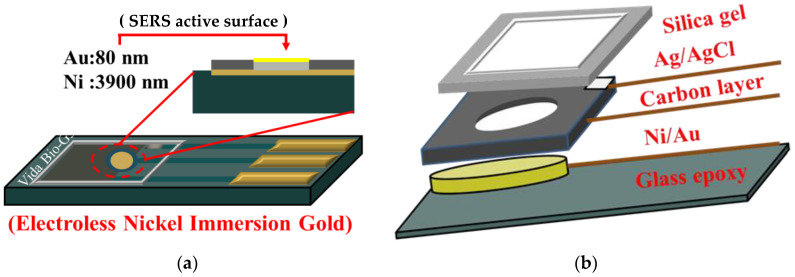
Screen-printed electrode—(**a**) oblique top view and (**b**) exploded view.

The substrate was a composite material composed of glass fiber woven cloth and a flame-retardant self-extinguishing epoxy resin binder. It was a screen-printed substrate made through three-pass screen-printing, plate-printed electrodes (including all wires, counter electrodes, and reference electrodes), and electroless nickel immersion gold was used to make the working electrodes. Silicone was finally put on to block the reaction area. The structure is shown in Figure 1.

### 2.3. Electrochemical Workstation

Our experiments were carried out on electrochemical workstations purchased from VidaBio. We used the modes of cyclic voltammetry (CV) and potentiostatic for experiments.

### 2.4. Observation and Analysis

An atomic force microscope (AFM) and scanning electron microscope (SEM) were used to analyze the surface morphology of our SERS substrate. The model of AFM and SEM were FM-1000 of Utek Material Co., Ltd. and JSM-6701F of JEOL Co., Ltd. Raman spectroscopy was used to measure the Raman signal of analytes on SERS substrates and the model of the instrument was SR500i of CL Technology Co., Ltd. The Raman microscopy system included a diode laser at 532 nm and a 50× objective lens. The SERS measurement was performed under an exposure time of 3 s and an accumulation number of 5 times by illumination with a 15 mW laser.

### 2.5. Experimental Procedure

**Figure 2:**
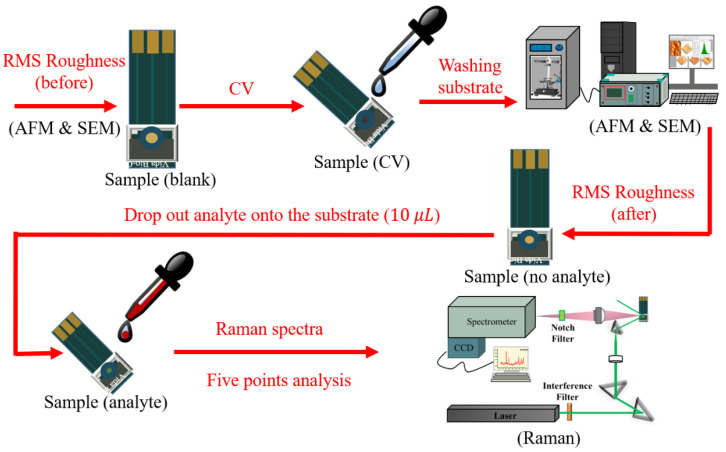
The flowchart of experimental procedure. AFM: atomic force microscope; SFM: scanning electron microscope; CV: cyclic voltammetry; SERS: surface-enhanced Raman scattering.

All experimental procedures are shown in Figure 2. After preparing the screen-printed electrode, we treated them by electrochemical oxidation-reduction cycles. Different concentrations of sulfuric acid (0.5 M, 0.25 M, and 0.1 M) were chosen as the electrolyte solutions. For each concentration of sulfuric acid, we used three different CV cycles (3, 6, and 9) to modify the surface roughness at a fixed sweep rate of 10 mV/sec. In this way, a total of 9 sets of experimental parameters were used to find the optimal substrate. The surface roughness, root mean square (RMS) deviation and morphology were measured and observed by AFM and SEM. The structure of the treated-substrate was a nature-patterned substrate with gold films over nanostructures and so-called NPS-AuFON.

After fabricating the substrate, R6G was used as a standard analyte to test the SERS effect and detection limit. Then, the above-mentioned substrate with optimized SERS effect analyzed by R6G was used to find the concentration limit for glucose detection. The goal was to obtain a better local surface plasmon resonance effect, and the final roughened substrate can detect low concentrations of glucose. To understand the reaction mechanism, one needs to understand the behavior of element deposition and examine the composition of the surface of the SERS substrate.

## 3. Results and Discussion

### 3.1. SERS Signals of R6G on the NPS-AuFON

**Figure 3:**
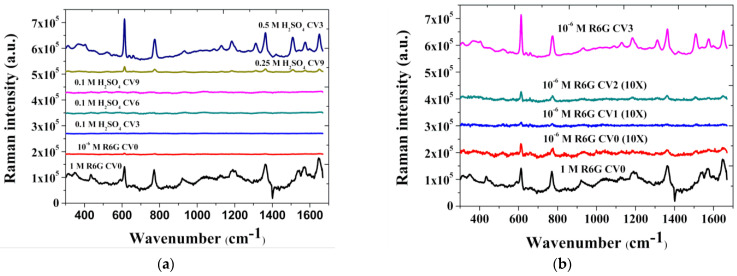
The intensity of the 10^−6^ M Rhodamine 6G (R6G) surface-enhanced Raman scattering (SERS) signal on the nanostructures (NPS-AuFON) under different concentrations of electrolytes and CVs—(**a**) CV 3 to CV 9; (**b**) CV 0 to CV 3.

In this study, a simple method was proposed to make a nature-patterned gold film for the substrate. Different concentrations of sulfuric acid (0.1 M, 0.25 M, and 0.5 M) and different CVs (0, 3, 6, and 9) with a fixed sweep rate (10 mV/sec) were used to change the surface roughness for enhancing the surface plasmon resonance to achieve the SERS effect. Next, 10^−6^ M R6G was used to test the SERS effect under different conditions. The results are shown in Figure 3a. R6G is a highly fluorescent dye; as such, there is an enormous baseline signal owing to its’ fluorescence. Therefore, we normalized the data. The result of those experimental conditions was that a sulfuric acid concentration of 0.5 M and a CV of 3 cycles (blue line) led to the best SERS effect for R6G detection. The Raman intensity and signal-to-noise ratio (S/N ratio) of the Raman peak was even better than the result of 1 M R6G detection on a non-roughened substrate (black line). Other conditions also had SERS enhancement, but the effect was not as obvious as the condition of 0.5 M and CV 3. However, although the operating condition with the best SERS gain effect were found in the foregoing, in order to understand the state of surface formation, and study the reasons for it, we fixed the concentration of sulfuric acid at 0.5 M and then scanned the redox cycles from CV 0 to CV 3. The result is shown in Figure 3b. The (10×) marked in the figure means that the signal was magnified 10 times.

**Figure 4:**
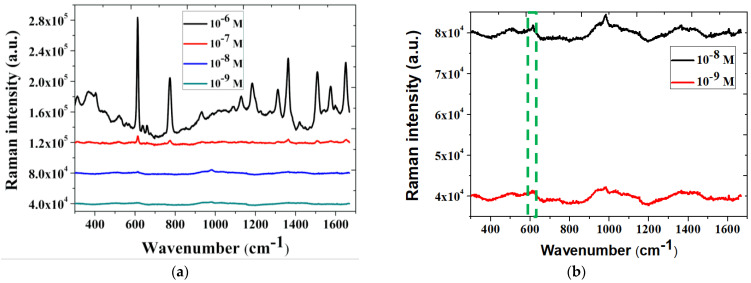
(**a**) The detection limit of R6G on the NPS-AuFON. (**b**) Zoom-in view of the spectra for low concentration conditions.

We observed that the Raman signal increases as the number of scanning cycles increase from CV 0 to CV 3 (Figure 3b). This confirms that the substrate processed by our method did not require any complex process of arraying patterned nanoparticles or SAM and still presented excellent SERS effect, amplifying the Raman signal of R6G. Looking into the fact that the increase in the number of CV cycles could enhance the SERS effect, we rationally inferred that the SERS enhancement mechanism was related to the surface roughening. In the follow-up, we used AFM and SEM for further analysis of the surface morphology on the processed substrate. After finding the best condition for SERS, we went further to determine the detection limit of R6G as shown in the Figure 4. We started from the R6G concentration of 10^−6^ M then decreased the concentration. According to the experimental results, using our NPS-AuFON substrate, the lowest concentration of R6G could be detected at about 10^−9^ M. We used the following formula [31] to calculate the SERS EF, which has been widely used to show the SERS effect. Therefore, we used the same definition to calculate the gain effect as shown Equation (1):

(1) $$EF=\left(N_{2}/N_{1}\right)\times \left(I_{SERS}/I_{ref}\right)$$

where $I_{SERS}$ and $I_{ref}$ are the intensities of SERS and normal Raman scattering, respectively. Here, we chose the Raman peak position at 614 cm^−1^ as the benchmark for comparison and calculation. This peak represents the C-C-C in-plane vibration mode of R6G [31]. $N_{1}$ is the number of molecules adsorbed onto the SERS substrate in the area being probed. $N_{2}$ is the number of molecules in the excitation volume of the laser used in normal Raman measurements. *N*_1_ is equal to the collection area divided by the surface area of one R6G molecule, and *N*_2_ is equal to irradiated solution volume multiplied by the concentration of analyzed molecules. The surface area of a single R6G molecule is 2 × 10^−18^ m^2^ [31]. The detailed calculation formulas are shown below.

(2) $$N_{1}=5.3\;\left({\mu m}^{2}\right)/2\times 10^{-18}({\;m}^{2\;})=\;2.65\times 10^{6}$$

(3) $$N_{2}=10^{-6}\;\left(\mathrm{mol}/L\right)\times 10\;\left(\mathrm{uL}\right)\times 6.02\times 10^{23}=6\times 10^{12}$$

(4) $$\;\;\;\;\;G=\left(6\times 10^{12}/2.65\times 10^{6}\right)\times \left(141654/3416\right)\;=9\times 10^{7}$$

The final result of the SERS EF was about 9 × 10^7^, which was similar to other reports that used SAM and arrayed nano-substrates to conduct SERS measurements.

### 3.2. Surface Morphology of Nano-Gold Electrodes

**Figure 5:**
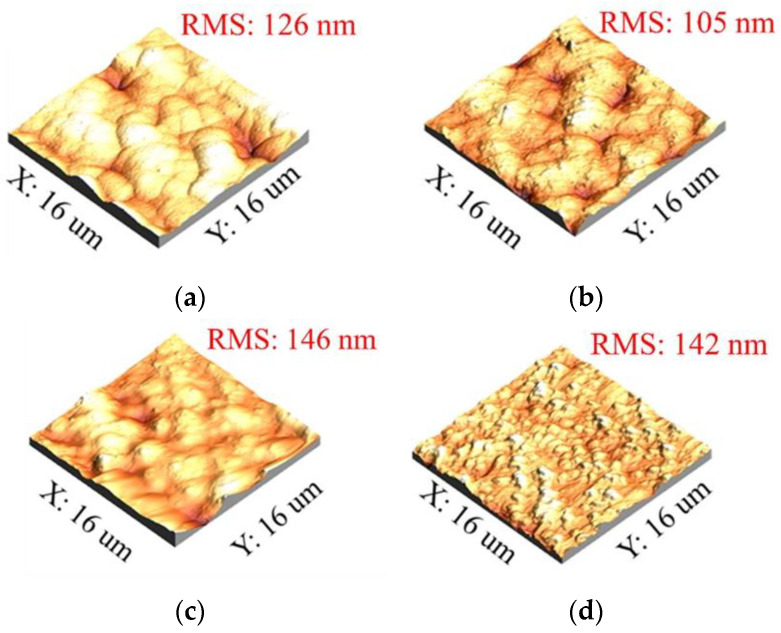
The roughness of the NPS-AuFON under different conditions. (**a**) Untreated; (**b**) CV 3 and 0.5 M H_2_SO_4_; (**c**) CV 9 and 0.25 M H_2_SO_4_; (**d**) CV 6 and 0.1 M H_2_SO_4_.

In a further analysis, AFM and SEM were used to observe the morphology of the NPS-AuFON surface. Figure 5 is an AFM scan image. We compared the untreated substrate surface and the substrate surface treated with three different parameters. The three different parameters were selected from the results of the best R6G signal obtained with different CVs and concentrations of sulfuric acid. From the scanning results, it was found that, except when the condition was 0.5 M sulfuric acid concentration and the scanning circle was CV 3, the surface roughness of other substrates was very large regardless of whether they were processed or not. Only with the condition of 0.5 M sulfuric acid and CV 3 could the surface roughness of the substrate manufactured reach a level close to 100 nm. At the same time, the substrate manufactured under this condition also had the best Raman signal during measurement. This was consistent with the conclusions reported in the literature, i.e., when the size of the gold nanoparticle on the substrate surface is 100 nm [15], the SERS substrate has the highest SERS EF. Here, we inferred that when the number of scanning cycles was small at the beginning, it indicated that the gold nanostructures began to form by an electrochemical micro-etching reaction. When the number of scanning cycles reached CV 3, the size of the gold nanoparticle reached the optimized 100 nm. When the number of scanning cycles increased again, the reaction became more intense. The surface etching phenomenon became more pronounced, and the surface roughness increased, which caused the size of the nanoparticles to be too large, and the SERS effect decreased.

**Figure 6:**
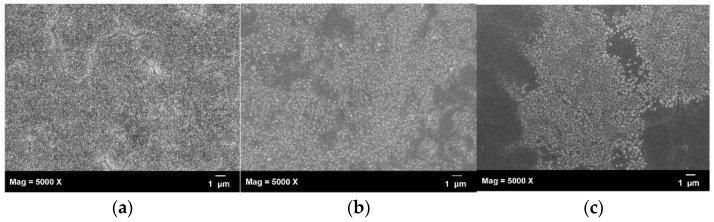
SEM images of the NPS-AuFON under different conditions—(**a**) CV 2, (**b**) CV 3, and (**c**) CV 6.

The above inference was confirmed by SEM observation, as shown in Figure 6. It was further confirmed that the surface roughness came from the formation of gold nanoparticles. Figure 6a–c show the surface of the substrate after two, three, and six scan cycles, respectively. We found that when the number of scanning cycles was small, the surface had just started to generate nano-gold particles. However, their size was not large enough and caused its SERS effect to be poor. As the number of cycles increased to three turns, it was clearly observed from the SEM images that many gold nanoparticles with a size of about 100 nm were generated on the treated surface. We estimated that this would have the strongest gain effect, which was consistent with the following Raman measurement results. After the CVs treatment exceeded 3 cycles, the surface nano-gold particles changed in size and began to aggregate with each other, this resulted in some areas exposed with the underlying metal pad due to over-etching, as shown in the grey part of Figure 6c. As the coverage area of the gold nanoparticle was reduced, the SERS effect of the substrate was also reduced.

### 3.3. SERS Intensity of Glucose on the NPS-AuFON

**Figure 7:**
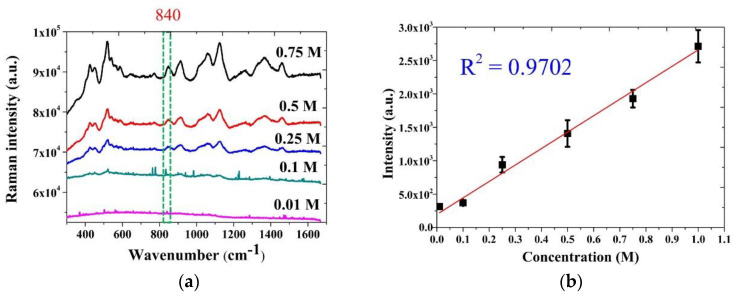
(**a**) The intensity of the Raman signals of different glucose concentrations on the NPS-AuFON. (**b**) The relationship between the Raman intensity and glucose concentration.

After finding the optimal condition for the NPS-AuFON, we used this condition to further detect different concentrations of glucose. The Raman signal spectrum is shown in Figure 7a, and the results confirmed that the detection limit of the substrate for glucose solution reached 0.01 M, which met the standards required by the WHO. Next, we chose the peak of 840 cm^−1^ as the comparison benchmark and plotted the calibration curve by the Raman signal intensity and the glucose solution concentration. The result is shown in Figure 7b. It was found that there was a good linear relationship between the two, and its R square value was as high as 0.97, indicating that the NPS-AuFON developed by our research team had a high quantitative analysis capability for these glucose solution concentrations.

### 3.4. Adsorption Behavior of Glucose on the NPS-AuFON

**Figure 8:**
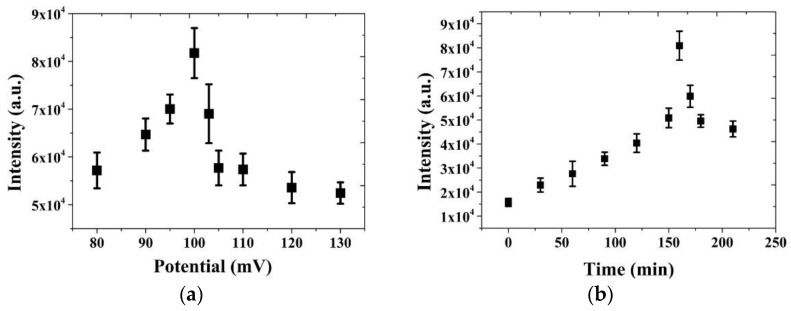
Electrochemical potentiostatic scan of 0.5 M glucose in 0.5 M sodium hydroxide solution. (**a**) The relationship between the Raman intensity and electric potential. (**b**) The relationship between the Raman intensity and reaction time.

After confirming that glucose could be effectively detected on the NPS-AuFON surface, we further sought to obtain the best detection conditions, and hoped to achieve those by adopting the method of electrochemical adsorption. Here, we used the potentiostatic method to carry out the follow-up experiments via different potentials and different reaction times as control variables and observed the changes in the SERS gain effect of the glucose solution. The results are shown in Figure 8a,b. From the experimental results, we found that the strongest Raman signal intensity was obtained when the reaction voltage and time were set at 100 mV and 160 min, respectively.

**Figure 9:**
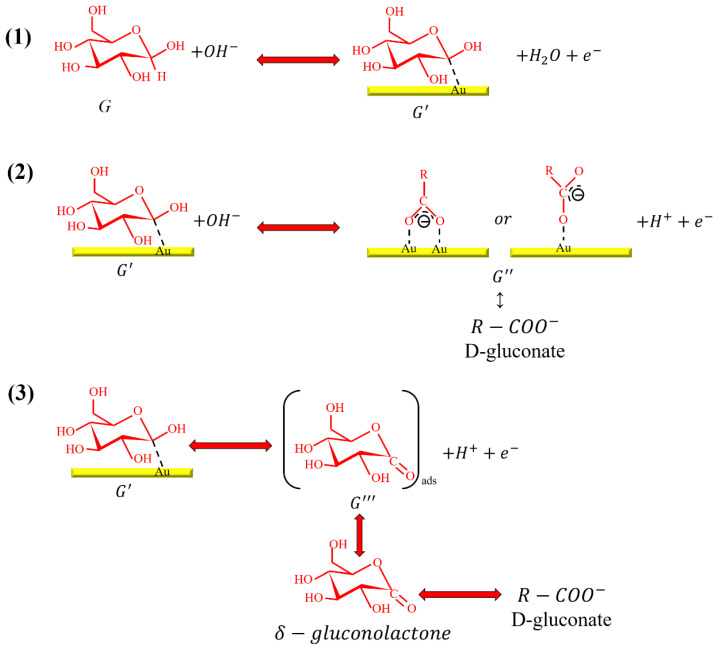
The schematic diagram of the reaction mechanisms of catalyzed glucose oxidation onto a gold surface. (1) Glucose is attached to gold electrode in alkaline solution. (2) To form D-gluconate by R-COO^-^ bond (3) To transform D-gluconate to δ-gluconolactone.

It was found from the above experimental results that different experimental conditions led to different SERS gain effects. We wanted to explain this phenomenon with a chemical reaction mechanism diagram [32]. In this experiment, glucose was dissolved into NaOH solution. When the glucose solution undergoes an electrochemical reaction there are three possible reaction mechanisms, as shown in Figure 9, of which the first reaction is the most crucial. This was also the main reaction of the glucose electrochemical adsorption proposed by our team. The glucose molecules were adsorbed in the alkaline solution on the surface of the gold electrode, then reactions (2) and (3) took place. Therefore, we observed that hydroxide (OH^-^) ion in the alkaline environment played a critical role in glucose adsorption. In other words, the entire electrochemical system was regarded as a pH dependent for the glucose oxidation reaction. The result of this electrochemical adsorption behavior further affected the Raman SERS signals.

## 4. Conclusions

We developed an innovative, low-cost, and easy-to-manufacture glucose measurement system in this research. The NPS-AuFON was fabricated by a low-cost screen-printed electrode using an electrochemical cyclic voltammetry. We found the optimum parameters by changing the electrolyte concentration and the number of CVs. In our experiments we found that by increasing the electrolyte concentration from 0.1 M to 0.5 M and reducing the number of CVs from 9 to 3 cycles, the surface roughness of the printed electrodes and uniformity of the deposited nano-gold improved. Next, we used AFM and SEM to observe the surface morphology of the substrate. From the SEM photos, we clearly observed the irregularly arranged gold nanoparticles, the size of which were about 100 nm. The AFM measurement further shows that the best SERS effect occurred when the surface roughness was about 105 nm.

We used R6G to verify the NPS-AuFON developed by our team and found that the detection limit of the R6G concentration was 10^−9^ M. Through calculations, the SERS EF reached 9 × 10^7^. Next, we detected glucose and obtained a linear relationship between its concentration and the Raman signal intensity. This measurement system reached the minimum detection limit standard set by the WHO regulations (11.1 mmol/L). At the same time, we used electrochemical adsorption technology to replace SAM for grabbing glucose to the electrode metal surface. By adopting the potentiostatic technique, we used a reaction potential of 100 mV and a reaction time of 160 min as the best parameters to obtain the glucose capture effect. We obtained the best SERS gain of the glucose Raman signal. We described the mechanism of the electrochemical adsorption reaction of glucose on the gold electrode in detail. Therefore, this study obtained a good SERS substrate through a simple CV process combined with electrochemical adsorption and printed electrode processes. The substrate, which is based on NPS-AuFON as the main structure and does not require an additional SAM, is suitable for qualitative and quantitative analysis of glucose.

## Data Availability

All data are contained within the article.
